# Chronic Administration of Anti-Stroke Herbal Medicine TongLuoJiuNao Reduces Amyloidogenic Processing of Amyloid Precursor Protein in a Mouse Model of Alzheimer’s Disease

**DOI:** 10.1371/journal.pone.0058181

**Published:** 2013-03-05

**Authors:** Ping He, Pengtao Li, Qian Hua, Yuan Liu, Matthias Staufenbiel, Rena Li, Yong Shen

**Affiliations:** 1 Center for Advanced Therapeutic Strategies of Brain Disorders, Roskamp Institute, Sarasota, Florida, United States of America; 2 Department of Pathology, School of Preclinical Medicine, Beijing University of Chinese Medicine, Beijing, China; 3 Novartis Pharm Ltd., Nervous System Research, Basel, Switzerland; 4 Center for Hormone Advanced Science and Education, Roskamp Institute, Sarasota, Florida, United States of America; Massachusetts General Hospital, United States of America

## Abstract

Composed of Ginsenoside Rg1 and Geniposide, the herbal medicine TongLuoJiuNao (TLJN) injection liquid has anti-inflammatory properties and can improve learning and memory in mice. Recently, TLJN has been used to treat the patients with cerebral ischemic stroke and vascular dementia, which significantly increase the risk of developing Alzheimer’s disease (AD) in the early human beings. Although beneficial effects of TLJN have been reported in the vascular-associated brain disorders, the roles of TLJN in AD brains are still not clear. In this study, we chronically administered TLJN in amyloid precursor protein (APP) Swedish mutant transgenic mice (APP23) from 6 months old of age, which is at the onset of Aβ plaques, to 12 months old. We found that TLJN significantly decreased Aβ production and deposition in the brain of APP23 mice. Furthermore, we observed that TLJN down-regulated the levels and activity of β-secretase 1 (BACE1) protein as well as the expression levels of γ-secretase complex components: PS1, nicastrin and anterior pharynx-defective 1 (APH1) but not presenilin enhancer 2 (PEN2). The results suggest an inhibitory effect of TLJN on amyloidogenic APP processing by down-regulating the cleavage enzymes BACE1 and γ-secretase.

## Introduction

TongLuoJiuNao (TLJN) injection liquid is an herbal medicine which is primarily composed of two active components: Ginsenoside Rg1 and Geniposide [1], [2]. Nowadays, TLJN has been used in the treatment of patients with cerebral ischemic stroke and vascular dementia [3], [4]. Ginsenosides belong to the class of steroid glycosides and triterpene saponins in the plant genus *Panax* (ginseng), which can suppress inflammation by nuclear factor κB (NF-κB) pathway [5], [6] and tumor growth by inhibiting DNA polymerase activity [7], [8]. Recent studies showed that Ginsenoside Rg1 could improve spatial learning and memory in rat models of Alzheimer’s disease (AD) [9], [10]. Another compound Geniposide in TLJN is an iridoid glycoside with a variety of biological activities including neuroprotection, anti-proliferation, and anti-oxidative stress [11], [12]. Besides the beneficial roles of TLJN in acute ischemic stroke and vascular dementia [3], [4], whether this anti-stroke herbal medicine could also be applied in the prevention and therapy of other neurological disorders such as AD is unknown.

AD is a neurodegenerative disease and pathologically characterized by excessive extracellular accumulation of amyloid β peptide (Aβ) in brains [13], [14]. Aβ is generated from the cleavage of amyloid precursor protein (APP) by two enzymes: β-secretase 1 (BACE1) and γ-secretase [15], [16]. Emerging evidence has shown that BACE1 expression levels and/or activities are increased in the brain of AD patients [17], [18], [19], [20]. Herbal medicines have been introduced to alleviate demented symptoms of AD patients [21], [22]. It has been suggested that Ginsenoside protects neurons against oxidative stress [23] and improves learning and memory functions [24], [25]. Experimental studies showed that administration of Ginsenoside significantly reduced Aβ levels in brains of Tg2576 mice, an AD mouse model [26] and senescence-accerlerated mouse prone 8 (SAMP8) mice [10], [27]. Furthermore, Ginsenoside is found to inhibit BACE1 activity by 80% in PC12 cells [28] and mouse neuroblastoma N2a cells expressing mutation human APP696 [29]. A recent report showed that TLJN increased the expression levels of IDE and NEP which both are involved in Aβ clearance [1].

In the present study, we intraperitoneally injected herbal medicine TLJN once a day in amyloid precursor protein (APP) Swedish double mutation transgenic mice (APP23) for 6 months (a critical period for Aβ deposition) [30]. We found that chronic administration of TLJN significantly reduced Aβ production and deposition and down-regulated BACE1 expression and activity as well as the expression of γ-secretase complex. However, Aβ degradation enzymes, neprilysin (NEP) and insulin degradation enzyme (IDE) were not affected in the animal model.

## Materials and Methods

### Component Analysis of Herbal Medicine TongLuaoJiuNao

**Figure 1:**
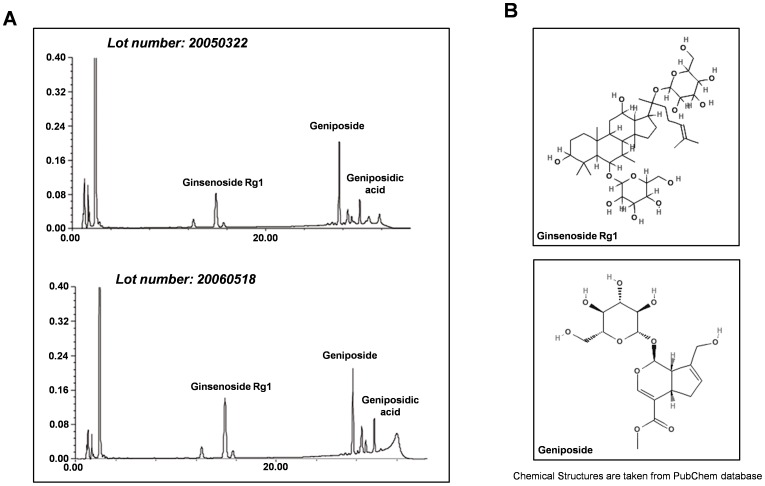
Chemical analysis of herbal medicine TongLuoJiuNao. (**A**) Ginsenoside and Geniposide in this study showed the same peak pattern. The figure was a representative of at least two separate experiments, each with triplicate samples. The chromatograms were scaled to the highest peak. (**B**) Chemical structure of Ginsenoside and Geniposide.

TLJN was purchased from Kang Yuan Pharmaceutical Engineering Limited Company, Neimenggu, China (Catalog: 051125). In recent years, the active components in TLJN were identified and widely applied in clinic and experiments [1], [2], [31]. In brief, the components are extracted from *Panax notoginseng* and *Gardenia jasminoides*. The compounds were processed and purified in accordance with the protocol of the National Medical Dictionary of China. TLJN was obtained by high-performance liquid chromatography (HPLC). The results of HPLC were showed in **Fig. 1A** and the chemical structures of the two components in **Fig. 1B**. The chemical analysis revealed Ginsenoside Rg1 as C_42_H_72_O_14_, molecular weight: 800 and Geniposide as C_17_H_24_O_10_, molecular weight: 300 [1]. The final concentration of the compounds was measured by HPLC with Ginsenoside Rg1 (1.02 mg/ml) and Geniposide (4.95 mg/ml) [1], [2], [31].

### Animals

All animal experiments were performed in compliance with a protocol approved by Roskamp Institute. APP23 transgenic genotypes (20 males in each group) in our experiments are on the C57BL/6 background, which are provided by Novartis Institute for Biomedical Research and the mice express mutated human βAPP (Swedish double mutation, KM670/671NL) under neuron-specific murine Thy-1 promoter element [32], [33]. Non-transgenic wild type and APP23 mice were crossed and the progenies were genotyped and characterized as APP23 with PCR followed by Western blot for confirming brain holoAPP protein. The resulting littermates were used in experiments [30], [34].

### Herbal Medicine TLJN Administration

The concentration used for the experiments was based on the reference of daily dose given to patients in clinical practice [1], [31]. For the observation of long-term effects on AD-like pathological changes, TLJN was administered from the age of 6th to 12th month (total six months). Mice were intraperitoneally injected once a day either with a dose of 300 µl herbal medicine TLJN solution per mouse (dilution of stock solution 1∶2 distilled saline, n = 20) or with the same volume of distilled saline as vehicle groups (n = 20). This dose (0.1 mg of Genisenoside Rg1 and 0.5 mg of Geniposide) of TLJN per mouse here was demonstrated without potential side effects observed in long-term treatment [1], [2], [31]. The treatment protocol for the herbal medicine is well tolerated by the animals. At the last day of the injection practice, mice were perfused with phosphate buffered saline (PBS) supplemented 10 U heparin. The brain tissues were dissected and the half of brain was post-fixed with 4% paraformaldehyde (PFA) for histological analysis and another half of the brain was stored in −80°C for biochemical analysis.

### Aβ Enzyme-linked Immunosorbent Assay

Enzyme-linked immunosorbent assay (ELISA) quantification was performed as described previously [34], [35], [36]. The APP23 mice in the presence (n = 10) and absence of TLJN (n = 10) were used for the determination of Aβ40 and Aβ42 levels. The neocortex tissues were isolated and homogenized in M-PER mammalian protein extraction reagent (catalog: 78503, Thermo Fisher Scientific) and centrifuged at 14,000×g for 20 min at 4°C. Protein concentrations were measured by protein assays (Bio-Rad Laboratories, Hercules, CA) following manufacturer’s instructions. The remaining pellet with insoluble Aβ was dissolved in 98% of formic acid and centrifuged at 4°C for 30 min. The supernatant from the pellet was collected for the assay of insoluble Aβ40 and Aβ42. The amount of Aβ40 and Aβ42 was measured with an Aβ40 and Aβ42 ELISA kit (KHB3481 and KHB3544, Invitrogen Life Technologies, Carlsbad, CA). The ELISA system has been extensively applied and there is no cross-reactivity between Aβ40 and Aβ42 protein. The quantitation of insoluble Aβ ELISA measurement was normalized to corresponding tissue protein concentration. Data were showed as Mean ± SD of four experiments.

### BACE1 Activity Assay

An aliquot of neocortex tissue homogenates (n = 5 in each group) was further lysed with a lysis buffer described as previously [30], [34]. Briefly, the supernatants were collected and BACE1 enzymatic activity was analyzed by using synthetic peptide substrates containing BACE1 cleavage site (HK(DABSYL)-SEVNLDAEFRQ(LY)) (BVI Substrate, a Lucifer Yellow labeled peptide, Catalog: #565781, Millipore Life Sciences Research, Billerica, MA). BACE1 substrate was dissolved in DMSO and mixed with HAc buffer (100 mM HAc and 100 mM NaCl, pH 4.5). An equal amount of protein was mixed with 100 µl of substrate. The fluorescence intensity was measured kinetically with a microplate reader for 2 hrs at 5 min reading intervals (Bio-Rad Laboratories) at an excitation wavelength of 430 nm and an emission wavelength of 520 nm. The average velocities were calculated and relative velocities compared to vehicle samples (100%) were plotted.

### Western Blotting

Western blot was performed as described previously [34], [37]. The neocortex from APP23 mice in the presence and absence of TLJN (n = 5 in each group) was homogenized in M-PER mammalian protein extraction reagent (catalog: 78503, Thermo Fisher Scientific) supplemented with Halt protease and phosphatase inhibitor single-use cocktail (Catalog: 78442, Thermo Fisher Scientific). Total proteins were separated by SDS-PAGE gel and then transferred to nitrocellulose membrane (Catalog: 162-0115, Bio-Rad Laboratories) using wet transfer equipment for overnight at 90 mA (Bio-Rad Laboratories). The blots were probed with antibodies as follows: mouse monoclonal antibody against APP (1∶2000, clone: MABP21, catalog: #44–100, Invitrogen), mouse antibody against BACE1 ectodomain (1∶2000, Clone: 137612, MAB931, R&D Systems), rabbit antibody against BACE1 N-terminal amino acids 46–62 (1∶2000; MAB0681, Sigma-Aldrich, St. Louis, MO), mouse anti-soluble human APP β Swedish mutation (sAPPβ-sw, 1;1000, clone: 6A1, catalog: 10321, IBL-America, **Minneapolis, MN**), rabbit polyclonal NEP antibody (1∶2000, sc-9149, Santa Cruz Biotechnology, Santa Cruz, CA), rabbit polyclonal IDE antibody (1∶25000, PC730, Millipore), rabbit anti-anterior pharynx-defective 1 (APH1) polyclonal antibody (H2D, 1∶5000, kind gift by Dr. Yueming Li, Memorial Sloan-Kettering Cancer Center, New York), rabbit polyclonal antibody against nicastrin (1∶1000, PA1-758, Thermo Fisher Scientific), mouse antibody against presenilin 1 C-terminal loop (PS1, 1∶1000, MAB5232, Millipore), rabbit polyclonal antibody against presenilin enhancer 2 (PEN2) (1∶1000, clone: UD-1, PRB-560C, Covance), rat antibody against presenilin 1 N-terminal (1∶1000, clone: hPS1-NT, MAB1563, Millipore), and mouse anti-β-actin antibody (1∶5000, clone AC-15, A1978, Sigma-Aldrich). Secondary antibodies against mouse or rabbit IgG conjugated HRP were applied (SC-2004 and SC-2055; Santa Cruz Biotechnology). The membranes were developed with SuperSignal West Femto Maximum Sensitivity Substrate (Catalog: 34095, Thermo Fisher Scientific) and the chemiluminescent image signal was captured using ChemiDoc XRS (Bio-Rad Laboratories). Quantitatively, the densitometry of the protein amount was measured using Quantity One software (Version 4.6.0, Bio-Rad Laboratories). The ratio of targeting protein density versus corresponding β-actin signal was calculated and further compared to vehicle groups (as 100%). The final results were expressed as density folds of the experimental groups to that of vehicles, accordingly.

### Histology and Immunostaining

Immunostaining was performed as described previously [30], [34]. The treated mice (n = 6 in each group) were perfused using 0.1 M phosphate buffer. The half brain was post-fixed in 4% PFA. Brain sections were performed serially (30 µm). To observe the Aβ fibril aggregation, the sections were incubated in thioflavine S solution for 5 min (1∶5000, T1892, Sigma-Aldrich). To test the Aβ accumulation, the sections were treated with 1% H_2_O_2_ for 10 min and blocked with 10% horse serum for 30 min. Monoclonal antibody against Aβ amino acid sequence 1–17 (1∶2000, clone 6E10, MAB1560, Millipore) was incubated in 4°C overnight. Following the primary antibody, the biotinylated secondary antibody against mouse IgG was applied (1∶1,000; Vector Labs, Burlingame, CA) for 30 min. Then VECTASTAIN Elite ABC kit (Vector Labs) was applied for 30 min and DAB (3,3′-diaminobenzidine) peroxidase substrate kit (Vector Labs) for 2 min. Counter staining was performed with Mayer’s haematoxylin Solution (Sigma-Aldrich) for 1 min.

### Quantification of Staining Structures

The sagittal sections per interval of 400 µm were chosen as histological and immunological staining. The counting of plaque number was carried out by an experimenter blind to the study [34]. A microscope (BX63; Olympus Corporation, Tokyo, Japan) with a 10x N PLAN, 20x and 40x PL FLUOTAR was used. Digitized images were captured with a DEI-470 digital camera (Optronics, Goleta, CA) on an Olympus microscope (Olympus). MagnaFire software (version 2.1C; Optronics) was applied. All of the immunopositive structures on each section were counted with same parameter. In general, 9–11 sections through the hippocampus formation per mouse were calculated (n = 6 mice in each group). The number of staining positive structures was totalized and expressed per section.

### Statistical Analyses

Statistical analyses were performed as described previously [34], [37]. Results were expressed as Mean ± SD and analyzed using a software program (SPSS version 11.5.1; SPSS). Student’s *t* test was used as a comparison of two groups. The level of significance was *p*≤0.05.

## Results

### Herbal Medicine TLJN Reduces Aβ Levels and Deposition

**Figure 2:**
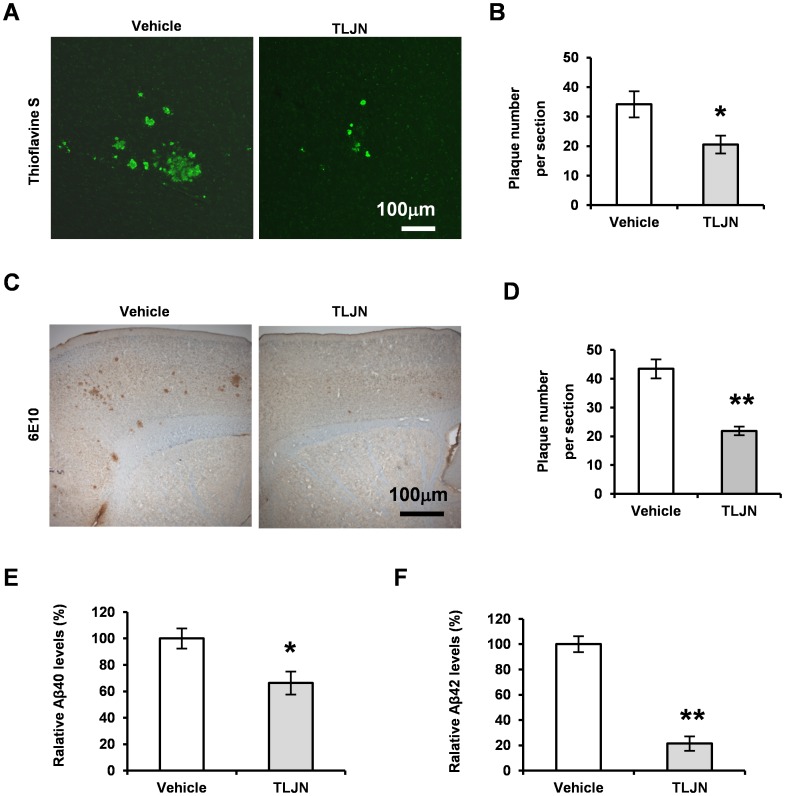
Herbal medicine TLJN reduces deposition and Aβ levels in APP23 mice. Representative images showed thioflavine S staining demonstrating insoluble Aβ deposition (**A**). The positive structures of thioflavine S histological staining were counted per section and statistical analysis showed a significant decrease in the number of plaques with TLJN treatment for 6 months (**B**, Mean ± SD, *p<0.05, Student *t*-test). Bar: 100 µm. Aβ accumulation was immunostained with 6E10 and microphotoimages were presented in (**C**). Similarly, 6E10-positive structures were counted and statistical analysis showed a significant decrease in the number of plaques in the brain treated with TLJN compared to vehicle groups (**D**, Mean ± SD, **p<0.01, Student *t*-test). Bar: 100 µm. ELISA was performed to measure insoluble Aβ40 and Aβ42 levels in the neocortex. Compared to vehicles, chronic treatment of TLJN significantly reduces Aβ40 (**E**) and Aβ42 levels (**F**) in the brain of APP23 mice (Mean ± SD, Student *t*-test, *p<0.05, **p<0.01).

Recent studies showed that Genisenosides could reduce Aβ42 levels in the brain of young Tg2576 mice [26]. At the age of 3–4 months old, there is not plaque formation yet in the brain of Tg2576 mice [38]. So, it is necessary to clarify whether Ginsenoside-induced reduction of Aβ levels is due to the decrease of insoluble Aβ aggregate in aged mice. To reveal the existence of plaques in the brain, the histological staining of thioflavine S, which binds to β sheet-rich fibril protein aggregates [39], was performed at the brain sections from the 12 months old APP23 mice in the presence and absence of TLJN (**Fig. 2A**). Results showed a significant reduction of thioflavine S positive deposits in TLJN-treated APP23 mice compared to vehicle groups (**Fig. 2B** **,** *p<0.05). A specific immunostaining identifying amyloid protein was also applied with antibody 6E10 recognizing Aβ1-17 fragment [40], [41]. Representative images of Aβ-positive staining plaques were shown in **Fig. 2C** **.** The number of all immuno-positive plaques was counted per section and a significant decrease in the number of Aβ deposits was observed following chronic TLJN administration (**Fig. 2D**, **p<0.05). These data showed that TLJN could reduce amyloid protein aggregation.

To further confirm whether the decreased Aβ deposition induced by herbal medicine TLJN administration in APP23 mouse brains was due to insoluble Aβ reduction, we measured the levels of insoluble Aβ40 and Aβ42 by sandwich ELISAs (n = 10 each group) [34], [36], which are primary Aβ species existing in amyloid plaques in AD brains [13], [14], [42], The pellets of detergent insoluble fractions from brain tissue homogenization were re-suspended with formic acid. Quantitative results showed that Aβ40 were significantly decreased by 34% in the subjects with TLJN chronic administration (2538±137 pg/mg) compared to the vehicle groups (3847±296 pg/mg) (Fig. 2E, *p<0.05). Insoluble Aβ42 levels were measured and observed a large decrease by 79% (893±32 pg/mg of in the presence of TLJN group vs 4192±381 pg/mg of the vehicle mice) (Fig. 2F, **p<0.01). These results suggest that TLJN could reduce Aβ levels, especially Aβ42 levels, consistent with previous report [26], and lead to the alleviation of Aβ pathology.

### Herbal Medicine TLJN Down-regulates BACE1 Activity and Levels

**Figure 3:**
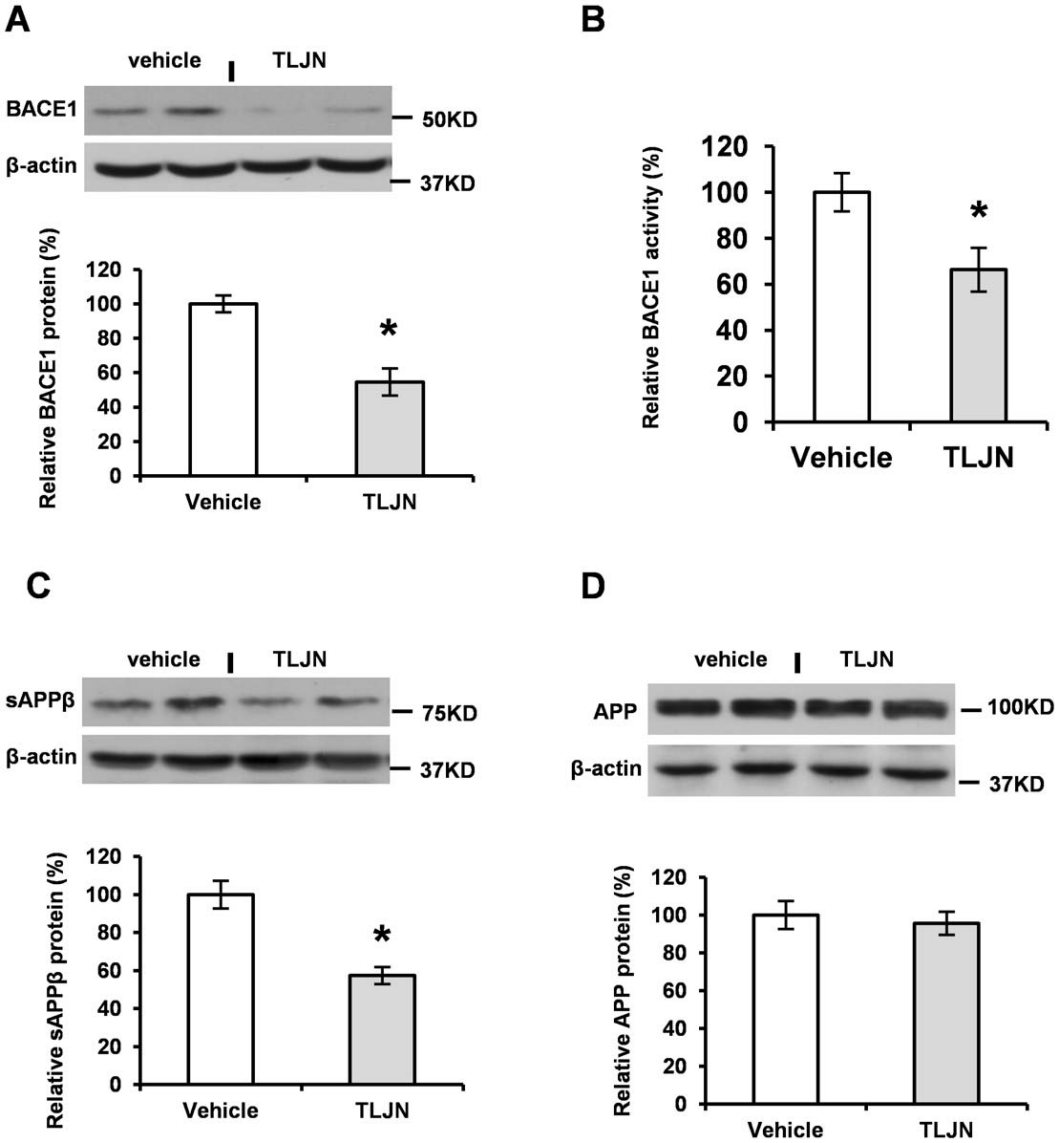
Herbal medicine TLJN down-regulates BACE1 levels and activity. BACE1 levels were probed by Western blot and its levels were normalized to β-actin level, accordingly (**A**). BACE1 activity was measured and relative activity levels were compared to vehicle controls (**B**, Mean ± SD, student t-test, *p<0.05). Soluble APP β fragment (sAPPβ) (**C**) and APP expression (**D**) were probed by Western blot and its levels were normalized to β-actin level, accordingly. Spot density analysis was performed and a significant decrease was observed in BACE1 (**A**), sAPPβ (**C**) but not APP levels (**D**) in TLJN-treated mice compared to vehicles. Statistical analysis was performed and significance was presented as *p<0.05 (Mean ± SD, Student *t*-test).

BACE1 is an enzyme responsible for β-site APP cleavage to produce Aβ peptide [40], [43], [44], [45], [46]. We recently reported that BACE1 protein levels and activity are increased in sporadic AD brains [19], [20]. Since Aβ level is reduced by TLJN application, we postulate that BACE1 might be one of the targets of TLJN. We detected BACE1 levels by Western blot (**Fig. 3A** **)** and found out a significant decrease of BACE1 protein levels in the TLJN application groups compared to vehicle groups (**Fig. 3A**).

It has been reported that Gensenoside Rg1 could inhibit BACE1 activity *in vitro* [28], [29]. However, BACE1 activity has not been examined yet in the brain of APP transgenic mice treated with TLJN. Following the administration of TLJN for 6 months, the BACE1 activity was then examined. Quantification showed that BACE1 activity is significantly decreased in TLJN-treated groups compared to vehicles (**Fig. 3B**). These results suggest that in addition to reducing BACE1 protein levels, TLJN could also decrease BACE1 activity. Proteolytic enzyme BACE1 cleaves APP to produce a soluble N-terminal fragment APPβ (sAPPβ) [13], [47]. To further verify the significant down-regulation of BACE1 by TLJN, Western blot were applied to test the secretion levels of sAPPβ fragments (**Fig. 3C**). We found a significant decrease of sAPPβ levels in the TLJN-treated APP23 mice (**Fig. 3C** **,** *p<0.05). To exclude the possibility that sAPPβ level decrease is due to the level reduction of substrate APP, APP was probed by Western blot. Results showed that there were no significant changes of APP protein levels in the groups between TLJN administration and vehicles (**Fig. 3D**), suggesting that TLJN down-regulates BACE1 but not APP expression in the brain of APP23 mice.

### Herbal Medicine TLJN Reduces Expression Levels of γ-secretase Components

**Figure 4:**
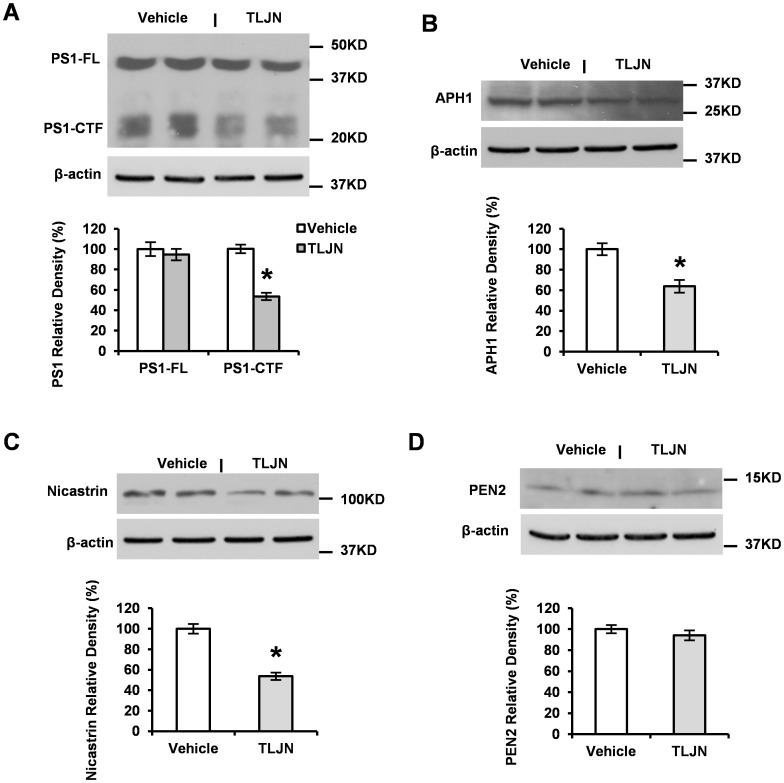
Herbal medicine TLJN reduces levels of γ-secretase components. APP23 mice were treated with TLJN for 6 months. Levels of γ-secretase components were analyzed by Western blotting. Representative images showed the bands of both PS1-CTF and PS1-FL (**A**), APH1 (**B**), nicastrin (**C**) and PEN2 (**D**). Then Spot density analysis was performed and a significant decrease was observed in PS1-CTF (**A**), APH1 (**B**) and nicastrin (**C**) but not in PS1-FL (**A**) and PEN2 (**D**). Significant differences were presented as *p<0.05 (Mean ± SD, Student *t*-test).

BACE1 cleavage generates a short C-terminal fragment (CTFβ) C99, which is then further processed by γ-secretase to produce Aβ peptide [16], [48]. The γ-secretase is a complex composed of four components: PS1, nicastrin, APH1 and PEN2. It has not been reported that TLJN could modulate the components of γ-secretase. Here, using Western blot, we detected the expression levels of γ-secretase complex in the presence and absence of TLJN. We found that TLJN did not affect full length PS1 but lowered PS1 C-terminal fragment levels (**Fig. 4A**). TLJN also reduced the levels of both APH1 (**Fig. 4B**) and nicastrin (**Fig. 4C**). However, we did not find significant changes of the component PEN2 protein expression (**Fig. 4D**). These results suggest that TLJN reduces Aβ production through modulating the levels of γ-secretase components.

### Herbal Medicine TLJN has Little Effect on Aβ Clearance Enzymes

**Figure 5:**
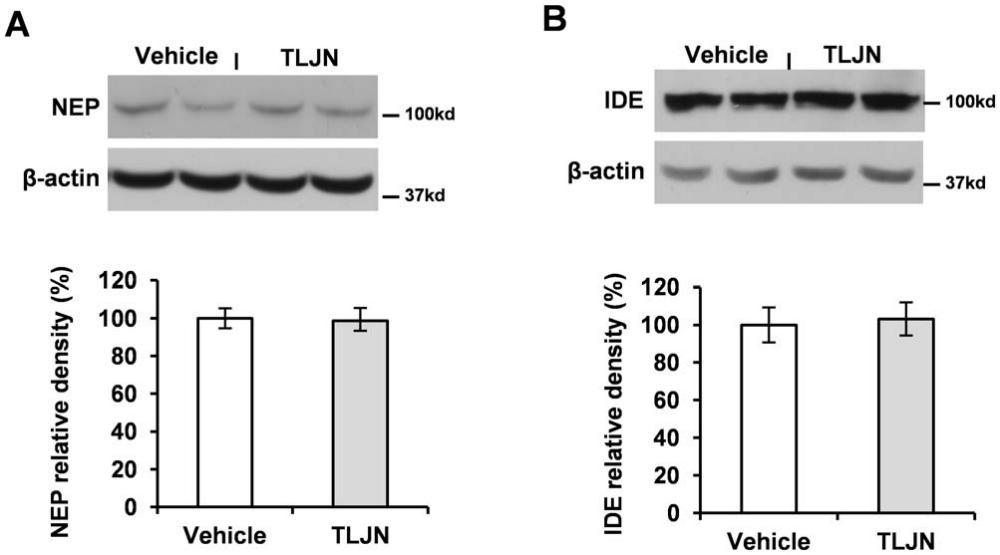
Herbal medicine TLJN has little effect on Aβ clearance enzyme expressions. APP23 were treated with TLJN for 6 months. Proteins from neocortex were loaded on an 8% Tris-Glycine gel. Membranes were blotted with rabbit polyclonal antibody against NEP (**A**) and antibody against IDE (**B**). Spot density analyses of NEP and IDE levels were performed and indicated as arbitrary units. No significant differences were presented as *p*>0.05 (Mean ± SD, Student *t*-test).

In addition to BACE1 and γ-secretase which are responsible for Aβ production, there are two enzymes relevant to Aβ degradation and clearance: NEP and IDE [49], which contribute to the degradation of Aβ levels in brains. Recent data showed that TLJN increases NEP and IDE protein levels in a rat model of AD induced by Aβ_25–35_ peptide intracerebral injection [1]. A recent report showed Geniposide could enhance IDE expression of cultured cortical neurons [50]. To examine the possibility that TLJN-induced Aβ reduction is also associated with Aβ degradation/or clearance in AD transgenic mice, we analyzed the levels of NEP and IDE in APP23 transgenic mouse brains with Western blotting. Unexpectedly, we did not find any significant elevation of either NEP (**Fig. 5A**) or IDE (**Fig. 5B**) levels following chronic TLJN administration in comparison to vehicle groups (n = 5 in each group).

## Discussion

TLJN is a modern Chinese formula that has recently been applied in phase II clinical trial in patients with cerebral ischemic stroke and vascular dementia [3], [4]. However, the roles of TLJN in AD still need to be tested in rigorous clinical trials. In the present study, we are the first to examine the effects of an anti-stroke herbal medicine TLJN on amyloid-related pathology in APP23 transgenic mice. Following TLJN chronic administration, we found in the brain tissues of APP23 mice that (1) Aβ levels and deposition were significantly decreased; (2) BACE1 protein levels as well as activity were significantly down-regulated; (3) the levels of γ-secretase components PS1, APH1 and nicastrin but not PEN2 were decreased; (4) there were not significant changes in the levels of the degrading enzymes NEP and IDE. Our finding of TLJN-decreased Aβ levels induced by TLJN is consistent with previous observations of Ginsenoside-induced Aβ level decrease [26], [27], [29], [51]. Therefore, Ginsenoside in TLJN might be the main active ingredients responsible for decreasing Aβ levels. In addition to Aβ reduction, we also found a significant decrease in the number of insoluble Aβ plaques in the chronic presence of TLJN. The number decrease may be associated with total reduction of Aβ levels. Meanwhile, there is another possibility that Ginsenoside in TLJN inhibits transforming of soluble Aβ to insoluble or directly blocks the aggregation of insoluble Aβ.

It has been reported that BACE1 is a stress-response protein [52], which means that its protein levels and/or activities might be modulated [53]. Here, we observed BACE1 level reduction in the brain treated with TLJN. In addition to TJLN-induced BACE1 protein level decrease, we also found that long-term TLJN treatment inhibited BACE1 activity in brains, consistent with the observation *in vitro* of BACE1 activity inhibition by the treatment of Ginsenoside Rg1 [28], [29]. The mechanism involved in BACE1 down-regulation by TLJN remains to be confirmed. It has been found that glucose reduction could possibly be involved in the early events of AD pathogenesis [54], [55]. It has been evidenced that glucose reduction as well as energy inhibition elevates BACE1 levels and activity in APP transgenic mice [54], [56]. In contrast, Ginsenoside stimulates glucose uptake [23]. Therefore, the capability of Ginsenoside to improve glucose uptake could be a cause of the regulation of BACE1 level and activity by TLJN in the brain of APP23 mice. Ginsenoside has also been implicated in down-regulating NF-κB signaling, resulting in anti-inflammatory consequences [57], [58], [59]. Combining our previous finding that genetic inactivation of tumor necrosis factor receptor-mediated NF-κB signal transduction pathway results in the decrease of BACE1 levels, ultimately leads to Aβ reduction [34], hence we postulate that TLJN-containing Ginsenoside down-regulates NF-κB signaling, resulting BACE1 inhibition. Recently, Ginsenoside has been found to enhance the binding of peroxisome proliferator-activated receptor γ (PPARγ) to BACE1 promoter, which in turn inhibits the transcription and translation of BACE1 and thus suppress BACE1 activity [29].

Another finding is that TLJN can partially decrease γ-secretase activity by reducing PS1, APH1 and nicastrin levels, which are components of γ-secretase enzyme complex. A number of small molecule γ-secretase inhibitors have been identified to block Aβ generation with high potency in cultured cells and in APP transgenic mouse models of AD [60], [61], [62]. However, It is noticed that γ-secretase also cleaves a number of additional substrates besides APP, such as Notch [48], [63] and ErbB-4 [64], and thus γ-secretase inhibitors can suppress Notch processing [65], [66]. Consequently, γ-secretase inhibition can result in serious adverse effects. Therefore, further elucidation of the mechanism of TLJN’s action is highly desirable for pharmacological intervention in AD.

One recent report suggested that TLJN improves Aβ clearance enzyme IDE and NEP expression [1]. It has further been demonstrated that Geniposide has the capability to up-regulate IDE expression in cultured cortical neurons [50]. However, we did not find a significant elevation of either IDE or NEP levels in the brain chronically treated with TLJN. The previous observation of elevating IDE and NEP levels is based on a rat AD model induced by bilateral intra-hippocampal injection of Aβ peptide 25–35. The discrepancy of these results may be due to different AD models used.

Neuron injury and loss is an important cause of dementia in AD patients. Another compound in TLJN is Geniposide, which has biological activities of neuroprotection and anti-oxidative stress. Lines of evidence have shown that Geniposide promotes nuclear translocation of nuclear factor-E2-related factor 2 (Nrf2) [11]. Binding of Nrf2 to the antioxidant response element induces a battery of gene transcriptions that can coordinate a protective response against oxidative stressors [67]. Geniposide also up-regulates the expression of heme oxygenase-1 (HO-1) [68]. HO-1 is an enzyme which is responsible for degrading heme to carbon monoxide, free iron, and biliverdin and for the cell defense against oxidative stress [69]. There has been evidenced that Geniposide could enhance insulin secretion [70], which prevents oxidative stress-induced damage in AD [71]. Recently, it has been found that Geniposide could inhibit Aβ42-mediated cytotoxicity in neurons [50]. Therefore, the roles of Geniposide in neuroprotection might be one of the beneficial actions of TLJN.

In summary, besides the application in the patients with cerebral ischemic stroke [2], [31], [72], we have identified anti-stroke herbal medicine TLJN as a modulator of BACE1 and γ-secretase complex, which provides a mechanistic clue for the application in neurodegenerative diseases such as AD.
